# Supplementary material for: SyNDI: synchronous network data integration framework
Source: BMC Bioinformatics. 2018 Nov 6;19:403. doi: 10.1186/s12859-018-2426-5 (PMC6219086; doi:10.1186/s12859-018-2426-5)
Supplement: Supplementary file 5 — Files of the example presented in Additional file 4. This additional file is a zip package that contains all files used in Additional file 4. Detailed descriptions of each of these files are presented in a README file included in this zip package. (GZ 30411 kb) [file 12859_2018_2426_MOESM5_ESM.gz › SyNDITools/Meme2Fimo/meme_4.9.0_4.tar.gz!/meme_4.9.0/doc/godag-format.html]

GO DAG Format


# GO DAG file format

The GO DAG file format is used by `gomo_highlight`
and `gomo`.
It is a quick parsing file, containing the structure and IDs from the Gene Ontology and can be
automatically generated from the
Gene Ontology's OBO format.
A GO DAG file is packaged with the gomo databases avaliable on the meme website.

---

## File Structure

The file structure can be divided into 3 major parts.

1. Header Comments
2. Directed Acyclic Graph
3. Graph Labels

### Header Comments

The purpose of the header comments is to document the details of the OBO file that was used to generate it.
Each line starts with a # symbol and the rest of the line can contain any content.

# Generated from an OBO file with the details:  
# OBO Format Version: 1.2  
# OBO Date: Fri Sep 25 11:40:00 EST 2009  
# OBO Saved By: jane  
# OBO Autogenerated By: OBO-Edit 2.1-beta1  
# OBO Remark: cvs version: $Revision: 1.804 $

### Directed Acyclic Graph

The purpose of the directed acyclic graph portion is to store the hierarchical structure of the
Gene Ontology in a way that is quick to load into memory while still being compact in file size.
The first line is the number of nodes in the DAG. Following that lines are in groups of 5 defining
different attributes of the node. The group is on its own in the first line. The name is on the second
line with the length coming first so memory can be preallocated for it. The node's position in the
DAG is summarized in the third line with the total number of nodes above followed by the total number
or nodes below. The fourth and fifth lines define the edges from the node to its parents and edges
from the node to its children. Each line of edges starts with the edge count for that line which can
be zero. Each edge is a number in the range zero to the node count minus 1 and is the index of the
linked node. Values on the same line are tab seperated. The order that the nodes are listed does
not have any meaning other than providing a position to for the other nodes to link to.

node\_count  
node\_1\_group  
node\_1\_name\_length         **tab** node\_1\_name  
node\_1\_nodes\_above         **tab** node\_1\_nodes\_below  
node\_1\_parent\_edge\_count [ **tab** node\_1\_parent\_1 ( **tab** node\_1\_parent\_w )\* ]  
node\_1\_child\_edge\_count  [ **tab** node\_1\_child\_1  ( **tab** node\_1\_child\_x  )\* ]  
...  
node\_n\_group  
node\_n\_name\_length         **tab** node\_n\_name  
node\_n\_nodes\_above         **tab** node\_n\_nodes\_below  
node\_n\_parent\_edge\_count [ **tab** node\_n\_parent\_1 ( **tab** node\_n\_parent\_y )\* ]  
node\_n\_child\_edge\_count  [ **tab** node\_n\_child\_1  ( **tab** node\_n\_child\_z  )\* ]

### Graph Labels

The purpose of the graph labels is to allow lookup of a graph node. Each line in the graph labels section has
a symbol indicating if the label is the primary (>) or alternate (+) label, followed by a tab, followed by the label,
followed by a tab, followed by the position of the associated node (index plus 1) or zero if the label is obsolete.
The labels are ordered alphabetically.

label\_count  
> **tab** label\_AA          **tab** position\_of\_node\_AA  
> **tab** label\_AB          **tab** position\_of\_node\_AB  
> **tab** label\_AC\_obsolete **tab** 0  
...  
+ **tab** label\_ZZ\_alt\_id   **tab** position\_of\_node\_AB

---

## Simple Example

Suppose there were the nodes A, B, C, D with the alternate names G,E,H,F and an obsolete name Z
all from the grouping 'example' which I will shorten to x.

| Node Name | Alternate Names | Parent Nodes | Child Nodes | Group | Nodes Above | Nodes Below |
| --- | --- | --- | --- | --- | --- | --- |
| A | G |  | B, D | x | 0 | 3 |
| B | E | A | C | x | 1 | 1 |
| C | H | B |  | x | 2 | 0 |
| D | F | A |  | x | 1 | 0 |

So one possible output for this example would be as follows. Note that this example
simulates tabs as tabs don't display properly in html.

# Simple example header  
4  

x  
35   The full descriptive name of node C  
2    0  
1    3  
0  

x  
46   Another descriptive name, this time for node A  
0    3  
0  
2    3    2  

x  
38   Meaningful descriptive name for node D  
1    0  
1    1  
0  

x  
21   Node B's name in full  
1    1  
1    1  
1    0  
9  
>    A    2  
>    B    4  
>    C    1  
>    D    3  
+    E    4  
+    F    3  
+    G    2  
+    H    1  
>    Z    0

---


## Generating from OBO files

If for some reason you can't source the GO DAG file from the meme website then the tool obo2dag
is provided in the scripts directory for the purpose of creating GO DAG files.
It is an executable jar file with source packaged in the jar. As the OBO file format is
still under active development we made use of a parser included with the
OBO-Edit program which means our program is
dependent on libraries from OBO-Edit. As this program is not likely to be needed by an end user
we have not sought permission to include OBO-Edit's parser and so you will have to source the
libraries yourself.

### Required Libraries for obo2dag

The tool obo2dag is dependent on the OBO-Edit classes:

- `org.obo.dataadapter.OBOParseEngine`
- `org.obo.dataadapter.OBOParseException`
- `org.obo.dataadapter.OBOParser`
- `org.obo.dataadapter.ParseEngine`
- `org.obo.datamodel.NestedValue`

These classes have their own dependancies and it was discovered through a process of trial and error
that the libraries needed from the OBO-Edit distribution are:

- obo.jar
- bbop.jar
- log4j-1.2.15.jar

The current distributions of OBO-Edit seem to only include automated installers (no tar version) and so
I recommend downloading the RPM version and using a program like 7-zip to extract the files you need.
I found that the jar files were in the rpm at the location "/./opt/OBO-Edit2/runtime/". Once you have
the jar files simply run obo2dag in the same folder.

### Running obo2dag

As stated previously, obo2dag is an executable jar file and so if your system is setup correctly you can
run it like you would a program and it will bring up the graphical user interface. To run the GUI from the
command line type:  
`java -jar obo2dag.jar`  
  
If you need to run obo2dag in non-GUI mode then you must specify the class `gomo.HierarchyParser`
and pass it the path to the obo file and the path to the output GO DAG file. The command is:  
`java -cp obo2dag.jar gomo.DAGParser <GO OBO File> <Output File>`
